# Supplementary figures and images for: Growth deficiency and enhanced basal immunity in Arabidopsis thaliana mutants of EDM2, EDM3 and IBM2 are genetically interlinked
Source: PLoS One. 2024 Feb 8;19(2):e0291705. doi: 10.1371/journal.pone.0291705 (PMC10852260; doi:10.1371/journal.pone.0291705)

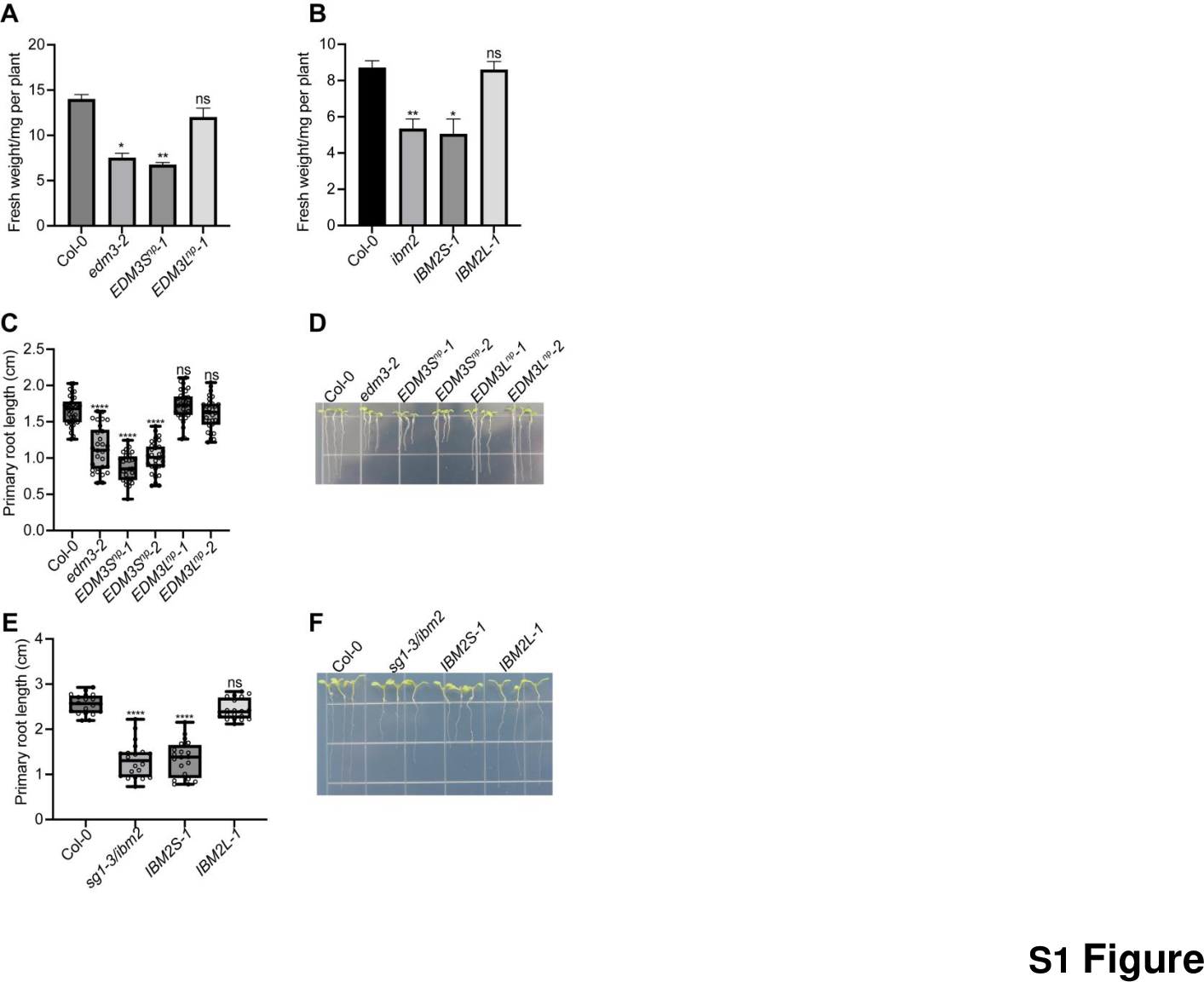

Supplement: S1 Fig — A Fresh weight of aerial parts from 15-day-old Col-0, edm3-2 or EDM3 isoform-specific complementation lines (EDM3Snp-1, EDM3Lnp-1) grown in soil. EDM3Snp-1 and EDM3Lnp-1 express in the edm3-2 mutant background either the short or long EDM3 isoform, respectively, driven by the native EDM3 promoter (np). B. Fresh weight of aerial parts from 12-day old Col-0, ibm2 and IBM2 isoform-specific complementation lines (IBMS-1, IBM2L-1) grown in soil. IBMS-1, IBM2L-1 express in the ibm2 mutant background either the short or long IBM2 isoform, respectively, driven by the native IBM2 promoter. The sg1-3 mutant allele of IBM2 was used for all experiments. C & E. Primary root length of 5-day-old plants of Col-0, edm3-2 and ibm2 plants as well as EDM3-and IBM2-isoform specific complementation lines grown on agar plates. D & F. Representative images of plants used in panels C and E. Data information: Error bars represent standard errors from three independent experiments. Asterisks indicate significant differences compared to Col-0 based on Student’s t-test. (*, p < 0.05; **, p < 0.01; ****, p < 0.0001; ns, no significance). n ≥ 28 (C) and n ≥ 20 (E). (TIF) [file pone.0291705.s001.tif]

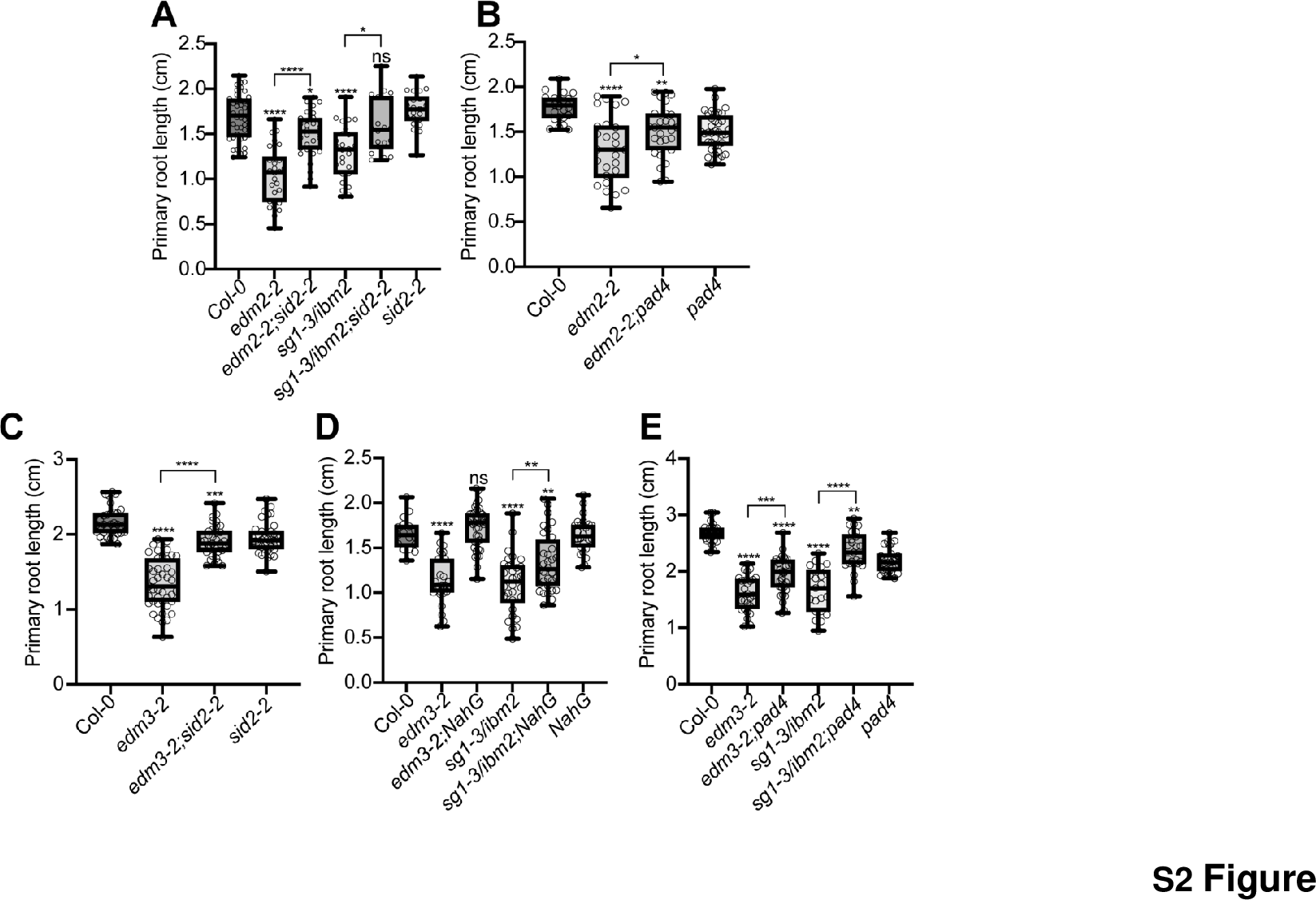

Supplement: S2 Fig — Primary root length of the indicated genotypes was measured using ImageJ. Data information: Data shown in each separate panel were generated simultaneously. Asterisks indicate significant differences compared to Col-0 based on one-way ANOVA (A-E). (*, p < 0.05; **, p < 0.01; ***, p < 0.001; ****, p < 0.0001; ns, no significance). n ≥ 18 (A), n ≥ 24 (B), n ≥ 33 (C), n ≥ 17 (D), n ≥ 21 (E). (TIF) [file pone.0291705.s002.tif]

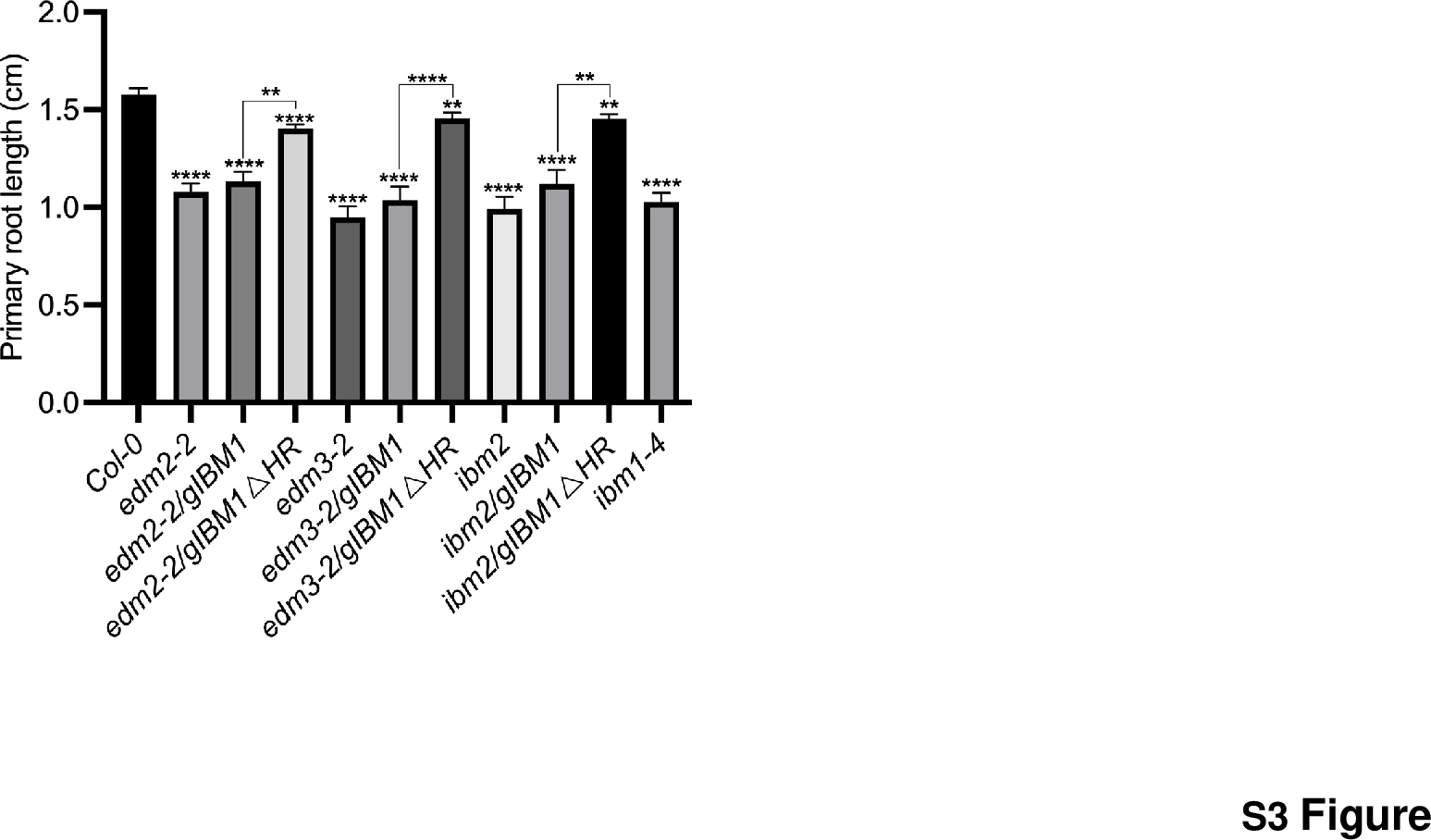

Supplement: S3 Fig — Primary root lengths of five day-old plants of the indicated genotypes were measured using ImageJ. Error bars represent standard errors. Asterisks indicate significant differences compared to Col-0 based on one-way ANOVA. (**, p < 0.01; ****, p < 0.0001). n ≥ 20. (TIF) [file pone.0291705.s003.tif]
