# Supplementary material for: Growth deficiency and enhanced basal immunity in Arabidopsis thaliana mutants of EDM2, EDM3 and IBM2 are genetically interlinked
Source: PLoS One. 2024 Feb 8;19(2):e0291705. doi: 10.1371/journal.pone.0291705 (PMC10852260; doi:10.1371/journal.pone.0291705)
Supplement: S1 Table — (DOCX) [file pone.0291705.s004.docx]

**S1 Table. Primers used in this study**

| Primer names | Sequence (5’-3’) | Notes |
| --- | --- | --- |
| PRX11_F | CGGACCAGGAGATGTACACG | Designed for this study. |
| PRX11_R | CCACTGGATCCTCCGCATAC | Designed for this study. |
| PRX15_F | CCAAGGTCTCGATCTCACCG | Designed for this study. |
| PRX15_R | TCTTCCAGCGCTGTTGATGT | Designed for this study. |
| PRX22_F | CCAGACCCAAGTCTGAACCC | Designed for this study. |
| PRX22_R | AAGCATCTGGAGTCACGACA | Designed for this study. |
| PRX25_F | ACCGGAAACTCCGATCCAAC | Designed for this study. |
| PRX25_R | TTCGAACCGTCTCCATTGGG | Designed for this study. |
| PRX27_F | AAAGGAGACAGCGACCCAAG | Designed for this study. |
| PRX27_R | CTAGAGCCGTCGTCGTATCG | Designed for this study. |
| PRX31_F | CGTCGCAACACGTGATCTTC | Designed for this study. |
| PRX31_R | CACCCGACCCACAAACTCTT | Designed for this study. |
| PRX49_F | AGCTCGTGGGTTTGACGTAG | Designed for this study. |
| PRX49_R | AGTGTTGTTTGGTGCAGGGA | Designed for this study. |
| PRX51_F | AGATGGACTTTCGTCGTCGG | Designed for this study. |
| PRX51_R | ATCCTAATGTGTGTGCCCCG | Designed for this study. |
| PRX53_F | TTTCGGCTGTCGGGCTTAAT | Designed for this study. |
| PRX53_R | GGATTTCCTGTCCCGCTGAA | Designed for this study. |
| PRX57_F | ACGGAAGCGTCAGGGAATTT | Designed for this study. |
| PRX57_R | CCAGAGACGGAGATCGTTGG | Designed for this study. |
| PRX62_F | GACTTTGTCCCCAAAACGGC | Designed for this study. |
| PRX62_R | GTCCCCGTCTTCACACCAAT | Designed for this study. |
| ACT8_F | CAGTGTCTGGATTGGTGGTTCTATC | [[1]](https://paperpile.com/c/jPDndt/B5xR) |
| ACT8_R | ATCCCGTCATGGAAACGATGT | [[1]](https://paperpile.com/c/jPDndt/B5xR) |
| RbohB_F | GTAAGTCGTTCGGTGCTATGT | [[2]](https://paperpile.com/c/jPDndt/gjSY) |
| RbohB_R | CAAACCTCATTGCAACCTCATC | [[2]](https://paperpile.com/c/jPDndt/gjSY) |
| RbohD_F | CCGGAGACGATTACCTGAGC | [[3]](https://paperpile.com/c/jPDndt/YqDu) |
| RbohD_R | CGTCGATAAGGACCTTCGGG | [[3]](https://paperpile.com/c/jPDndt/YqDu) |
| RbohF_F | CTTGGCATTGGTGCAACTCC | [[4]](https://paperpile.com/c/jPDndt/Z9Tp) |
| RbohF_R | TCTTTCGTCTTGGCGTGTCA | [[4]](https://paperpile.com/c/jPDndt/Z9Tp) |

1. [Tsuchiya T, Eulgem T. An alternative polyadenylation mechanism coopted to the Arabidopsis RPP7 gene through intronic retrotransposon domestication. Proc Natl Acad Sci U S A. 2013;110: E3535–43.](http://paperpile.com/b/jPDndt/B5xR)

2. [Mira MM, Huang S, Kapoor K, Hammond C, Hill RD, Stasolla C. Expression of Arabidopsis class 1 phytoglobin (AtPgb1) delays death and degradation of the root apical meristem during severe PEG-induced water deficit. J Exp Bot. 2017;68: 5653–5668.](http://paperpile.com/b/jPDndt/gjSY)

3. [Yeung E, van Veen H, Vashisht D, Sobral Paiva AL, Hummel M, Rankenberg T, et al. A stress recovery signaling network for enhanced flooding tolerance in Arabidopsis thaliana. Proc Natl Acad Sci U S A. 2018;115: E6085–E6094.](http://paperpile.com/b/jPDndt/YqDu)

4. [Morales J, Kadota Y, Zipfel C, Molina A, Torres M-A. The Arabidopsis NADPH oxidases RbohD and RbohF display differential expression patterns and contributions during plant immunity. J Exp Bot. 2016;67: 1663–1676.](http://paperpile.com/b/jPDndt/Z9Tp)
