# Supplementary material for: Growth deficiency and enhanced basal immunity in Arabidopsis thaliana mutants of EDM2, EDM3 and IBM2 are genetically interlinked
Source: PLoS One. 2024 Feb 8;19(2):e0291705. doi: 10.1371/journal.pone.0291705 (PMC10852260; doi:10.1371/journal.pone.0291705)
Supplement: Attachment — Submitted filename: Comments 26-09-2023.docx [file pone.0291705.s005.docx]

**General comments**

Overall, the research paper presents a thorough investigation into the genetic interplay between EDM2, EDM3, and IBM2 genes in Arabidopsis thaliana mutants. The study effectively addresses the defense-growth trade-off, a crucial aspect of plant immunity and development. The methodology is well-designed, incorporating a range of experimental techniques to explore genetic interactions and phenotypic outcomes. Overall, the study is well designed and executed. The following minor comments/suggestions should be addressed.

Stick to the Journal format of paper writing including sequence in the sections and references style

**Specific comments**

**Abstract**

Since you mention "We recently reported...", it would be helpful to briefly mention any key findings from your previous work to provide context for this study.

End the abstract with a clear statement summarizing the main takeaway or contribution of the research.

**Introduction**

When introducing terms like "NLR-type immune receptor" or "cytosine methylation," consider providing brief explanations or references for readers who might not be familiar with these concepts.

It might be helpful to clarify the full names of the genes (EDM2, EDM3, and IBM2) upon their first mention, even though you provide explanations later. This can enhance initial understanding for readers.

While it's implied, consider explicitly stating the hypothesis or research question that your study aims to address. Additionally, mention the specific objectives or goals of your research at the end of the introduction.

**Results**

The use of specific mutant alleles (sg1-3, edm2-2, edm3-2) is well-described. Providing these details is important for reproducibility and allows other researchers to understand the specific genetic context of your experiments.

The observation that edm2, edm3, and ibm2 mutants have shorter primary roots is a significant finding. when discussing the selection of type-III peroxidase genes, it might be helpful to briefly explain why PRX31 and PRX62 were not included. It would be interesting to speculate on the potential molecular mechanisms underlying this phenotype.

**Treatment Timing:**

The choice of treatment timing (day 14 and day 16) seems justified, but it would be helpful to provide a rationale for why these specific time points were chosen. This could be based on the growth stage or a previous study's findings.

**Dosage of Inhibitor:**

The concentration of the peroxidase inhibitor (20μM of SHAM) is specified. It would be beneficial to briefly explain why this particular concentration was chosen, especially if it aligns with prior research or known effective concentrations.

**Measurement of Basal Resistance**

Clarify how basal resistance is measured. This could involve details about the quantification or assessment of HpaNoco2 spores, including the method used and any potential controls or replicates.

**Rationale for Genomic Clones in IBM1L partly rescues growth defects and disease susceptibility in mutants of EDM2, EDM3 and IBM2:**

It would be helpful to provide a brief explanation of why these specific genomic clones were chosen and how they relate to the research question.
